# Chinese Customs during Menstruation

**Published:** 1865-12

**Authors:** 


### CHINESE CUSTOMS DURING MENSTRUATION.

At a meeting of the Edinburgh Obstetrical Society, Dr. Keiller stated that a naval officer, now stationed in China, had directed his (Dr. K.'s) attention to the peculiar manner in which the Chinese women were accustomed to treat themselves during menstruation. The gentleman referred to had, in his communication, enclosed a folded sheet of soft and apparently very bibulous paper, which he stated was similar to that generally used in China instead of the ordinary cloth or napkin used by females in this country. Dr. K. read from the letter he had received the following observations regarding the matter:

"You will be much surprised and puzzled when you receive this letter and its enclosure; so I will at once clear it up. I was talking to Dr. Medows, of Ningpo, about the personal cleanliness of the Chinese being so bad, when he told me it was not so bad as I supposed, and mentioned that in one particular they were more careful than ourselves; that is, with women during their menstrual period, instead of using a cloth as European women do, they use the paper enclosed, which is folded exactly as it is in the envelope, except that I have turned down one end to make it fit the envelope. He tells me a belt is used, and a cloth to go over the paper and keep it in place. The Chinese amas (servants) to European ladies refuse to wash the cloths which they use, and ladies therefore use the paper, and Dr. Medows tells me they prefer it. The Chinese always burn it. I do not know if such paper could be procured at home; but should you think it worth a trial in Scotland, I will be most happy to send you some, and make arrangements for your receiving a regular supply, should it be adopted to any extent, and no substitute found at home. I have troubled you with this because I believe small things contribute much to the comfort of all, and are more frequently overlooked because it is no one's business. I have so little to do of my own at present that I have to look out for some."—*Edinburgh Medical Journal.*

